# A Potential miRNA-mRNA Network for Dementia and Hernia Crosstalk

**DOI:** 10.1155/2021/4324068

**Published:** 2021-07-23

**Authors:** De-jian Chen, Da-peng Li

**Affiliations:** Department of Day Care (Hernia Center), Shanghai General Hospital, Shanghai Jiao Tong University School of Medicine, China

## Abstract

**Background:**

It has been reported that there may be a potential link between hernia and dementia. However, the exact mechanisms of their association have not been established. This study is aimed at constructing miRNA-mRNA networks to elucidate on the potential link between dementia and hernia.

**Methods:**

Gene expression profiles for dementia, herniation, and skeletal muscle were downloaded from the GEO database after which differentially expressed mRNAs and miRNAs were obtained. In addition, fascia tissue samples were obtained during surgery. A total of 41 patients were recruited in this study, and expression levels of candidate genes were examined using quantitative RT-PCR. Luciferase reporter gene assays were used to identify potential miRNA-mRNA regulatory pathways.

**Results:**

Differentially expressed mRNAs and miRNAs were screened. A potential miRNA-mRNA network revealing the crosstalk mechanism between herniation and dementia was identified. Single cell analysis revealed that PI16 was highly enriched in adipose tissues, skeletal muscles, and in the skin. GSEA enrichment analysis showed that PI16 is involved in adipose metabolism, muscle functions, and energy metabolism. In clinical samples, PI16 was found to be upregulated in hernia, while miR-4451 was found to be downregulated. The luciferase reporter gene assay revealed that downregulation of circulating miR-4451 may be responsible for the upregulated PI16 expression in hernia sacs.

**Conclusions:**

We constructed an miRNA-mRNA network that shows the potential association between dementia and hernia. We also found that miR-4451 regulates the PI16 expression, which may be a key target and biomarker for hernia pathogenesis and dementia crosstalk.

## 1. Introduction

Ageing is a global social issue, and the number of people with dementia is expected to reach 75 million by 2030 and could exceed 100 million by 2050 [1]. The prevalence of dementia is positively correlated with age and various diseases including cardiac failure, depression, dyslipidemia, chronic obstructive pulmonary disease, stroke, and head injury among others, which are risk factors for dementia [2–4]. Formation of an abdominal wall hernia may be associated with various factors. A previous cohort study reported that there may be a potential link between hernias and dementia 5. However, the possible mechanisms underlying the potential link between hernias and dementia have not been established.

MiRNAs regulate the gene expression by complementary pair binding to transcript regions of mRNAs. In some cognitive impairment-related diseases, expressions of miRNAs are altered [5, 6]. Based on microRNAs analysis, there is a link between age-related muscle atrophy and cognitive impairment [7]. Regulatory effects of microRNAs on muscles have been confirmed [8–10]. Older people with weak abdominal muscles and fascia are prone to hernia. In addition, changes in circulating microRNA levels are associated with development of congenital hernias [11]. Aberrant regulation of let-7b-5p, miR-1307-3p, miR-185-3p, miR-8084, miR-331-3p, and miR-210-3p may also contribute to the development of congenital diaphragmatic hernia [11]. Therefore, we hypothesized that some miRNAs are differentially expressed in the blood of dementia and hernia patients, and that these miRNAs could be markers for organismal weakness, acting as a link between dementia, muscle weakness, and hernia development.

This study is aimed at constructing miRNA-mRNA networks to elucidate on the potential link between dementia and hernia.

## 2. Materials and Methods

### 2.1. Microarray Data

To retrieve target gene expression profiles from the GEO dataset (http://www.ncbi.nlm.nih.gov/geo), we downloaded the hernia dataset GSE196374, the cognitive injury miRNA dataset GSE120584, and the skeletal muscle miRNA dataset GSE165632. The gene expression profile GSE19637 consisted of 17 samples from the skin tissue (8 from the normal tissue and 9 from the hernia tissue) and 17 samples from the fascia tissue (8 from the normal tissue and 9 from the hernia tissue). The gene expression dataset GSE120584 included 1021 sera samples from Alzheimer's disease patient sera and 288 sera samples from healthy people. The gene expression dataset GSE165632 included 5 samples from sedentary patients and 9 samples trained for long-term endurance and resistance. The full matrix file and sample information files were downloaded for further bioinformatic analysis.

### 2.2. Screening of DEMs and DE-miRNAs

Analysis of the difference in mRNA expression matrix was realized using the LIMMA package moderated paired *t*-test difference test [12]. Edge*R* package was used for differential analysis of miRNA expression matrix [13]. The plot function from the “base” package was used to plot the volcanoes and mark key mRNAs or miRNAs on the volcanoes. GO and KEGG enrichment analysis was then performed on the differentially expressed mRNAs (DEMs) obtained using the aforementioned methods. Bar graphs of the enrichment analysis were plotted (*p* < 0.05).

### 2.3. miRNA-mRNA Regulatory Network

The miRWalk3.0 database (http://mirwalk.umm.uni-heidelberg.de/), which includes 10 databases (RNA22, PITA, PICTAR5, PICTAR4, Targetscan, RNAhybrid, miRWalk, miRDB, miRanda, and DIANAmT), was used to construct the miRNA-mRNA regulatory network.

### 2.4. Functional Validation

Gene set enrichment analysis (GSEA) was performed based on the core mRNAs obtained from the screening. The GSEA software (version 4.1.0) was employed to perform GSEA enrichment analysis, and a random sample size of 1000, *p* < 0.05, was set as the threshold of statistical significance. Immunohistochemical data were obtained from the Human Protein Atlas (THPA) (https://www.proteinatlas.org/). The HPA is licensed under the Creative Commons Attribution-ShareAlike 3.0 International License [14].

### 2.5. Quantitative Real-Time PCR (qRT-PCR) and Sample Collection

A total of 41 patients were recruited for this study. Out of the total patients, 21 belonged to the hernia group while 20 belonged to the normal group. Ethical review and consent for this study were obtained from the Ethics Committee of Shanghai General Hospital. Fascia tissue samples were obtained during surgery. Isolation of total RNA from the tissues was done using the Mini-BEST Universal RNA Extraction Kit (TaKaRa, Kyoto, Japan) as guided by the manufacturer. Subsequently, RNA was converted to cDNA using reverse transcription with Prime-ScriptRT (TaKaRa). Then, the qPCR was performed on a Cycler 480 (Roche Diagnostics, Basel, Switzerland) using SYBR Green Master Mix (TaKaRa). In parallel, we isolated total miRNA from cell cultures using TRIzol® Reagent. The quantity and quality of miRNA were determined using stem-loop quantitative RT-PCR (TaqMan probe method). The first strand cDNA of purified MIRNA was synthesized using M-MLV reverse transcriptase and primers according to the instructions in the protocol provided by the manufacturer (Promega, Fitchberg, MA, USA). The relative gene expression was determined using the U6 gene as an internal standard. All qRT-PCR analyses were carried put in triplicates.

### 2.6. Dual Luciferase Reporter Gene Assay

The HEK293 cell line is a highly efficient and easy to culture cell line for transfection. The PI16 reporter plasmids were obtained from GeneChem (Shanghai, China). Cotransfection of the reporter vector with PI16-WT or PI16-Mut into HEK293 cells was conducted with the Lipofectamine 2000 system (Invitrogen, Carlsbad, CA, USA). Cotransfection of the reporter vector with PI16-WT or PI16-Mut into HEK293 cells was conducted with the Lipofectamine 2000 system (Invitrogen, Carlsbad, CA, USA) for 48 h. Thereafter, the luciferase reporter system was then used to assess luciferase activity.

### 2.7. Statistical Analysis and Gene Single Cell Expression Database

Statistical analyses were completed using *R* software (version 4.0.2) and GraphPad software (version 7.0). Differences between the two groups were compared by two-tailed Student's *t*-test (*p* < 0.05 was considered statistically different). Analysis of the gene expression within cells was completed based on previous studies [15]. Tabula Muris is a compilation of single cell transcriptional data from Mus musculus containing nearly 100,000 cells from 20 organs and tissues.

## 3. Results

### 3.1. Identification of DEMs and Enrichment Analysis

**Figure 1:**
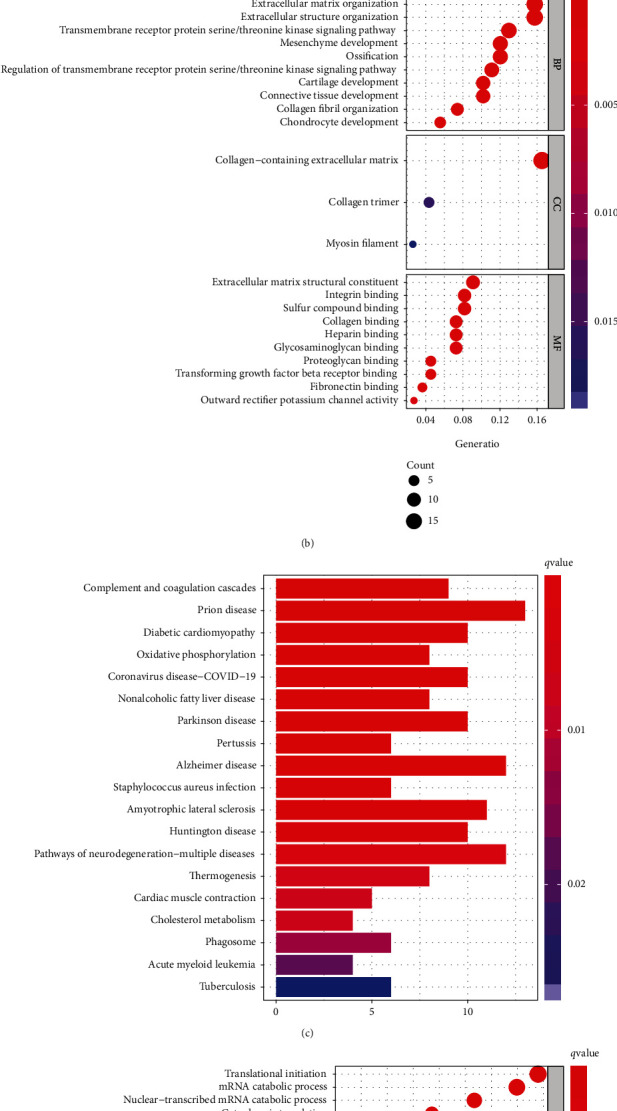
Enrichment analysis of DEMs. GO enrichment analysis of DEMs in the fascia tissue (a, b). KEGG enrichment analysis of DEMs in the fascia tissue (c). Enrichment analysis of DEMs in the skin tissue.

In the hernia dataset, GSE196374, we identified 148 genes and 123 genes that were upregulated in skin and fascia tissues where hernia occurred, respectively. In addition, 189 genes and 146 genes were downregulated in the skin and fascia tissues where hernias occurred, respectively. GO and KEGG enrichment analyses were performed for these up- and downregulated DEMs, respectively. GO enrichment analyses of DEMs in fascia tissues are shown in Figures 1(a) and 1(b). The KEGG enrichment analyses of DEMs in fascia tissues are shown in Figure 1(c). Enrichment analyses of DEMs in skin tissues are shown in Figure 1(d).

### 3.2. Identification of Co-DEMs and DE-miRNAs

**Figure 2:**
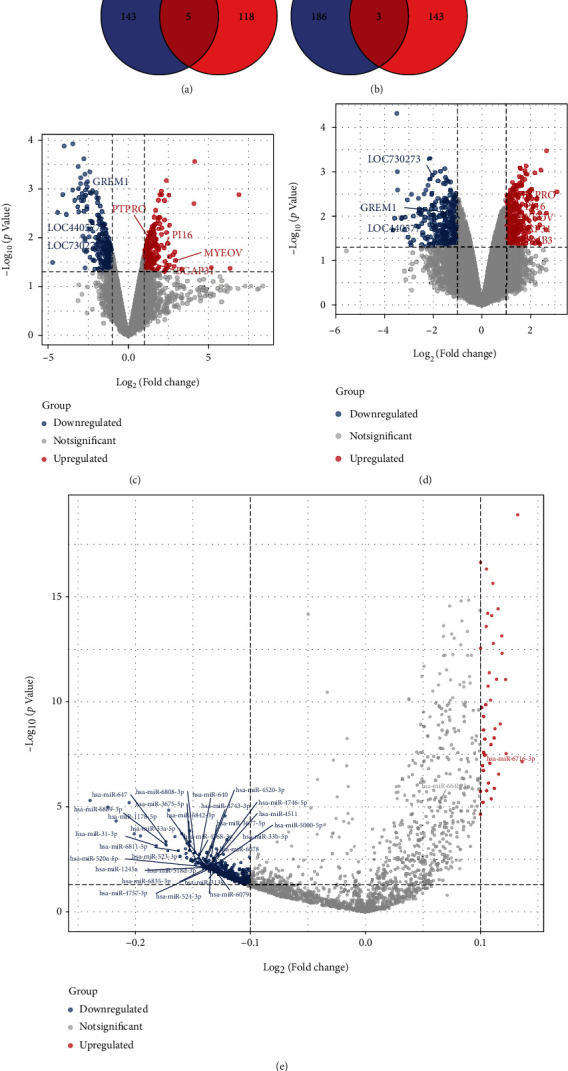
Identification of co-DEMs and DE-miRNAs. Veen plot intersection analysis identified mRNAs that were codifferentially expressed in hernia (both skin and fascia) (a, b). Volcano plots were used to demonstrate codifferentially expressed genes (c, d). Differentially expressed miRNAs were screened from the cognitive damage miRNA dataset (GSE120584) (e).

To identify mRNAs that are codifferentially expressed in hernia sacs (both skin and fasica), we performed veen plot intersection analysis (Figures 2(a) and 2(b)). These coexpressed differential genes were presented in a volcano plot (Figures 2(c) and 2(d)). We found that PTPTO, PI16, BCAP31, MYEOV, and PSMB3 were upregulated in the fascia and skin while LOC730273, GREM1, and LOC440577 were downregulated in the fascia and skin. Differentially expressed miRNAs (DE-miRNAs) in the cognitive impairment miRNA dataset GSE120584 were also screened out (Figure 2(e)).

### 3.3. Construction of the miRNA-mRNA Regulatory Network

**Figure 3:**
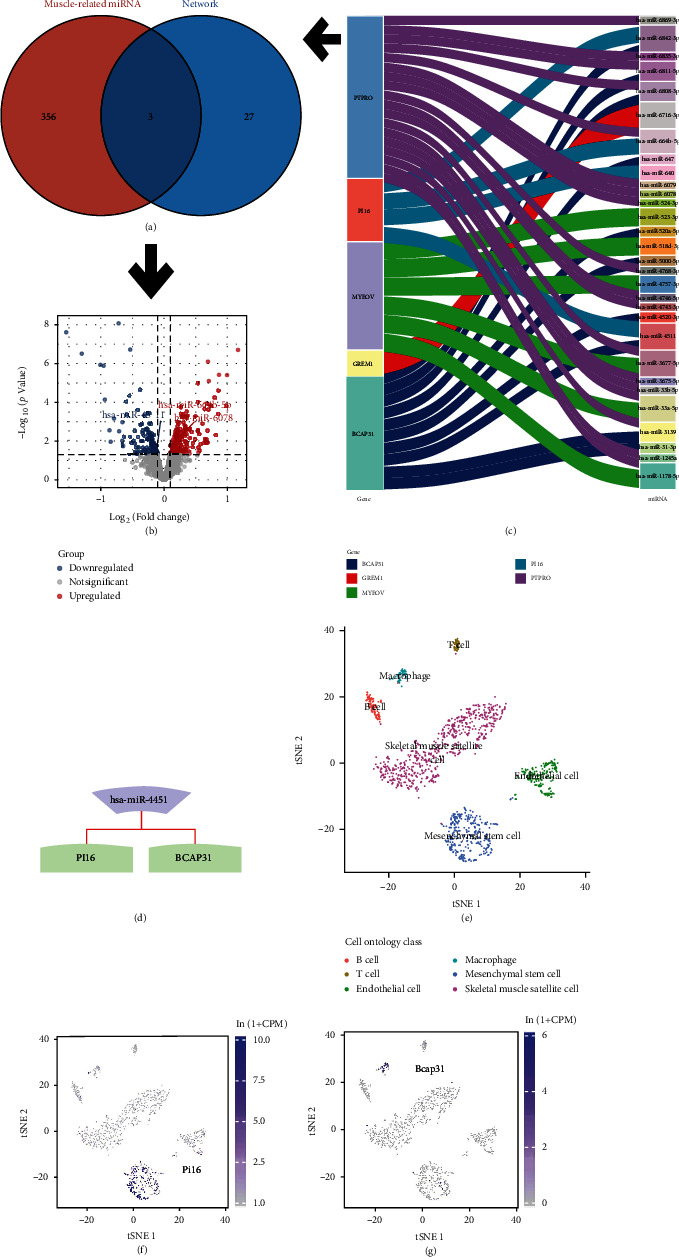
Construction of miRNA-mRNA regulatory networks and single-cell expression profiling. The intersections of 359 muscle-related miRNAs and 30 DE-miRNAs in the miRNA-mRNA network were determined by mapping Wayne plots (a). Volcano plot of intersecting genes (b). Sankey plots showing miRNA-mRNA networks constructed based on the miRWalk3.0 database (c). miR-4451 regulates PI16 and BCAP31 expressions (d). Annotated map of the single cell expression from mouse tissues (e). Pi16 was found to be highly enriched in mesenchymal stem cells (f). Bcap31 was found to be enriched in most cell types (g).

To construct the miRNA-mRNA regulatory network, we constructed a miRNA-mRNA network (Figure 3(c)) based on the miRWalk 3.0 database. The network is composed of DE-miRNAs and DEMs. A total of 30 DE-miRNAs and 5 mRNAs were included. The skeletal muscle miRNA dataset GSE165632 (samples from participants serum) was used to obtain 359 muscle-related miRNAs. These muscle-related miRNAs may be associated with abdominal wall muscle weakness. Three miRNAs (hsa-miR-4511, hsa-miR-664b-5p, and hsa-miR-6078) were obtained by plotting these 359 muscle-related miRNAs against 30 DE-miRNAs in the miRNA-mRNA network (Figure 3(a)). These results are presented in the volcano plot, as shown in Figure 3(b). Therefore, miR-4451 was found to be coassociated with muscle atrophy and cognitive ageing. Based on the results in Figures 3(c) and 3(d), hsa-miR-4451 is suggested as a possible sponge regulating PI16 and BCAP31.

### 3.4. Expression Distribution of Core mRNAs

**Figure 4:**
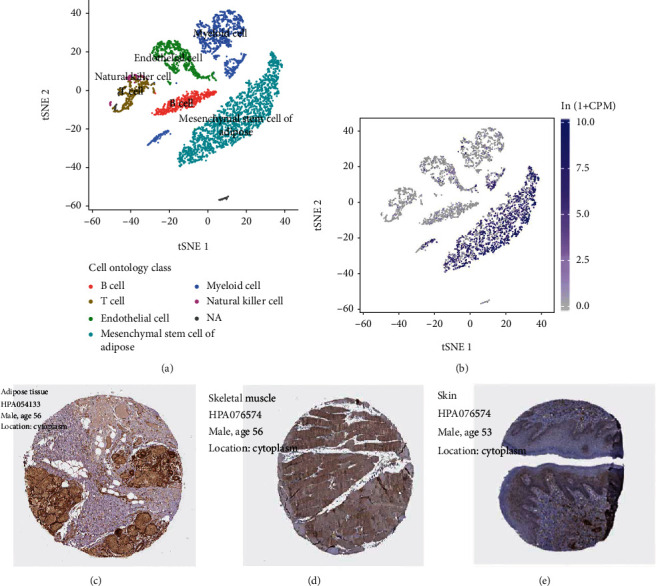
Expression of PI16 in the adipose tissue. In the adipose tissue, Pi16 was found to be enriched in mesenchymal stem cells of adipose (a, b). In the immunohistochemical profile of the THPA database, Pi16 was found to be widely enriched in the adipose tissue, skeletal muscle, and skin (c, d).

We analyzed the expression of Pi16 and Bcap31 in skeletal muscles at the single cell level based on Tabula Muris. The single cell expression annotation profile is shown in Figure 3(e). Pi16 was found to be predominantly enriched in mesenchymal stem cells (Figure 3(f)). Bcap31 was not found to be specifically enriched in any of the analyzed cell types (Figure 3(g)). In adipose tissues, Pi16 was found to be enriched in mesenchymal stem cells, whereas the Bcap31 expression was not detected (Figures 4(a) and 4(b)). Mesenchymal stem cells have an important role in skeletal muscle development, and therefore, we selected Pi16 for further analysis. Figures 4(c) and 4(d) show immunohistochemical profiles from the THPA database. Pi16 was found to be highly enriched in adipose tissues, in the skeletal muscle, and in the skin. In addition, Pi16 was also enriched in the cytoplasm.

### 3.5. Functional Characterization of PI16

**Figure 5:**
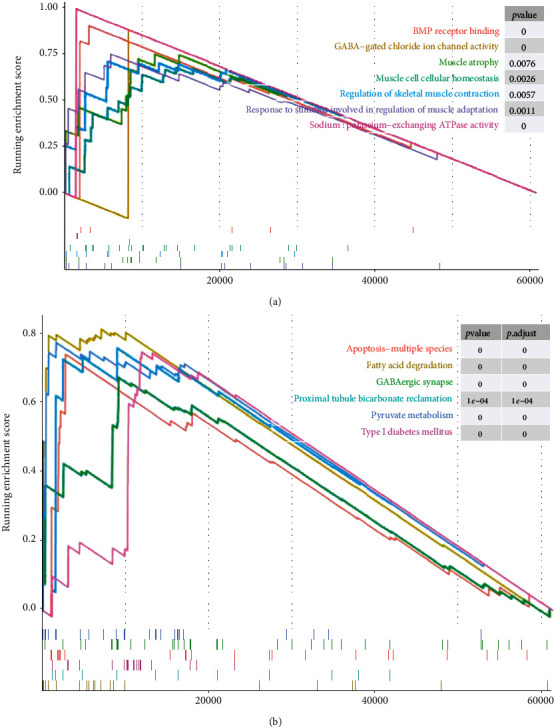
GSEA enrichment analysis showing GO functional analysis and KEGG pathway analysis associated with PI16. GO enrichment analysis (a). KEGG enrichment analysis (b).

GO functional enrichment analysis of GSEA revealed PI16-related signaling functions, including BMP receptor binding, GABA-gated chloride ion channel activity, muscle atrophy, muscle cell cellular homeostasis, regulation of skeletal muscle contraction, and response to stimuli involved in regulation of muscle adaptation (Figure 5(a)). In addition, KEGG enrichment analysis revealed the functions associated with PI16 isoforms, including apoptosis-multiple species, fatty acid degradation, GABAergic synapse, proximal tubule bicarbonate reclamation, pyruvate metabolism, and type I diabetes mellitus (Figure 5(b)). Enrichments of these functions and pathways implied that PI16 may play a role in fat metabolism, muscle function, and energy metabolism.

### 3.6. Cognitive Impairment Upregulates PI16 by Downregulating miRNA-4451 and Is Associated with Hernia Development

**Figure 6:**
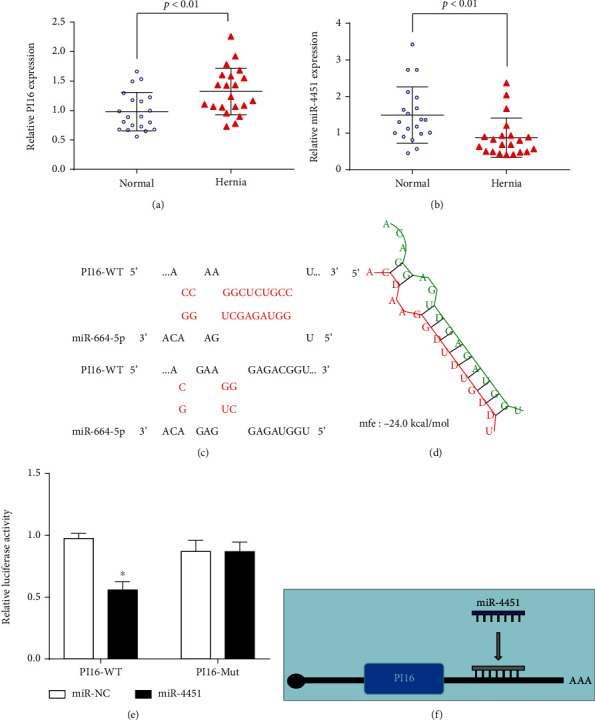
miR-4451 downregulates the expression of PI16. The relative expression of miR-4451 and PI16 in normal and hernia, respectively, was detected by qPCR (a, b). Pattern plots of PI16 wild type and miR-4451 binding with PI16 mutant groups (c). miR-4451 was determined as a target miRNA for PI16 using RNAhybrid 2.12. The minimum free energy was 24 kcal/mol (d). Expression levels of mir-32-3p in HEK-293 cells were detected by RT-qPCR after transfection of cells with mir-32-3p mimics (e). Pattern diagram of miR-4451 possibly acting as a sponge for PI16 and downregulating PI16 expression (f).

Expression levels of miR-4451 and PI16 in tissue samples were determined by qRT-PCR (Figures 6(a) and 6(b)). PI16 was found to be upregulated in hernia (*p* < 0.01) while miR-4451 was downregulated (*p* < 0.01). We hypothesized that miR-4451 may be involved in hernia pathogenesis by regulating PI16 transcription. To test this hypothesis, we predicted the potential binding site of miR-4451 to PI16 using RNAhybrid (Figure 6(d)). Luciferase reporter assays revealed that HEK-293 cells cotransfected with PLA2G4A-mut and the MIR-NC control had lower luciferase activity, while HEK-293 cells cotransfected with PI16-WT and miR-4451 had higher luciferase activities (Figures 6(c) and 6(e)). Moreover, miR-4451 acts as a sponge for PI16 to downregulate its expression (Figure 6(f)). We added Table 1 to present the lack of significant differences in clinical characteristics between patients who developed a hernia and those who did not. These findings imply that downregulation of circulating miR-4451 may be responsible for upregulated PI16 in hernia sacs.

## 4. Discussion

Weak abdominal muscles and fascia among elderly individuals predispose them to hernia. In this study, miRNAs, which are commonly associated with muscle atrophy and dementia, were identified, and the potential miRNA-mRNA regulatory networks were established. miR-4451 may inhibit PI16 transcription, which may be a potential link between herniation and dementia development. In conclusion, this study revealed a potential link between dementia and herniation at the molecular regulatory level.

We identified DEMs that are codifferentially expressed in the hernia dataset. These DEMs were also analyzed for enrichment. Then, the dementia miRNA dataset (GSE120584) and the muscle atrophy miRNA dataset (GSE165632) were combined to construct a potential miRNA-mRNA regulatory network. The Tabula Muris and THPA web databases were used to evaluate the distribution of hub mRNA in cells and tissues. Pi16 was found to be highly enriched in adipose tissues, in skeletal muscles, and in the skin. GSEA enrichment analysis revealed a possible role of P16 in adipose metabolism, muscle function, and energy metabolism. In clinical samples, PI16 was found to be upregulated in hernia, while miR-4451 was downregulated. The luciferase reporter gene assay showed that mR-4451 may act as a sponge for P116, downregulating the P116 expression. Therefore, downregulation of circulating miR-4451 may be responsible for upregulated PI16 in hernia sacs.

The peptidase inhibitor 16 (PI16) is a member of the CAP protein superfamily, a group of proteins that are evolutionarily highly conserved [16, 17]. PI16, a secretory protein that is mainly localized in the endoplasmic reticulum and secretory vesicles of fibroblasts, plays an important role in the cardiovascular system [18]. The overexpression of PI16 inhibits MMP activity, and MMPs, including MMP1, MMP2, MMP9, and MMP13, are associated with abdominal hernia development [19–22]. In this study, Pi16 was found to be highly enriched in adipose tissues, skeletal muscles, and in the skin. GSEA revealed that PI16 functions in fat metabolism, muscle, and energy metabolism. These findings confirm a previously documented hypothesis [23].

The potential of miR-4451 as a cancer biomarker has been reported [24–26]. We postulated that miR-4451 may be a biomarker for the development of hernia and cognitive impairment. Cognitive impairment may be associated with hernia development by downregulating miR-4451 to upregulate PI16. However, other biological roles of miR-4451 should be further investigated.

A previous cohort study found that there may be a potential link between hernias and dementia; although, the exact mechanism is unknown [23]. Bioinformatics is a powerful research method for predicting molecular mechanisms and associations between genes. We used bioinformatic approach to investigate the crosstalk between abdominal hernias and cognitive impairment. A potential miRNA-mRNA network for dementia and hernia was constructed. It was found that miR-4451 is involved in the regulation of the PI16 expression. This study is associated with some limitations, and therefore, caution should be exercised before referring to our findings. Further studies should include more factors and involve more clinical samples to determine the effect of miR-4451 on PI16. This is because the pathogenesis of abdominal hernias is complex, and alterations in abdominal muscles and adipose tissues are unlikely to be the only factors involved. The most significant DE-miRNAs and DEMs were the only ones included in this study. In future, we will perform animal and cellular experiments to elucidate on the mechanisms of miR-4451's action on PI16.

## 5. Conclusion

The constructed miRNA-mRNA network reveals the potential association between dementia and hernia. miR-4451 is involved in the regulation of the PI16 expression, which may be a key target and biomarker for evaluating hernia pathogenesis and dementia crosstalk.

## Figures and Tables

**Table 1:** Differences of clinical characteristics between effective And ineffective groups.

Characteristics	Normal [*N* = 20]	Hernia [*N* = 21]
Gender		
Female	11 [55%]	9 [43%]
Male	9 [45%]	12 [57%]
Age		
Mean [SD]	70.7 [7.8]	65.6 [5.5]
Median [Min, Max]	70 [60, 90]	64 [60, 81]
BMI		
Mean [SD]	22.7 [2.5]	24.7 [3.2]
Median [Min, Max]	22.8 [19.2, 29.1]	24.3 [18.5, 31.5]
Hypertension		
Yes	8 [40%]	8 [38%]
No	12 [60%]	13 [62%]
Diabetes		
Yes	4 [20.0%]	3 [14%]
No	16 [80%]	18 [86%]
Hyperlipidemia		
Yes	4 [20%]	6 [29%]
No	16 [80%]	15 [71%]

## Data Availability

If raw data is required, please contact the corresponding author for further access.

## Abbreviations

DEMs: Differentially expressed mRNAs
DE-miRNAs: Differentially expressed micro-RNAs
GO: Gene ontology
GEO: The Gene Expression Omnibus
GSEA: Gene-set enrichment analysis
KEGG: Kyoto Encyclopedia of Genes and Genomes
miRNA: Micro-RNA
NC: Normal control
qPCR: Quantitative real-time PCR.
